# Workout Logging Through an mHealth App for Weight Reduction Among Different Generations: Secondary Analysis of the MED PSU×ThaiSook Healthier Challenge

**DOI:** 10.2196/45298

**Published:** 2023-06-30

**Authors:** Kittiya Sae-lee, Decho Surangsrirat, Chanatip Parlawong, T-touch Anawilkul, Narinuch Assawachamrun, Pawanya Boonbandan, Paweerata Ladapongpuwat, Bhawat Chupetch, Supatcha Thongchai, Nannapat Pruphetkaew, Therdpong Thongseiratch, Polathep Vichitkunakorn, Pitchayanont Ngamchaliew

**Affiliations:** 1 Faculty of Medicine Prince of Songkhla University Hat Yai, Songkhla Thailand; 2 Assistive Technology and Medical Devices Research Center National Science and Technology Development Agency Bangkok Thailand; 3 Department of Epidemiology Faculty of Medicine Prince of Songkla University Hat Yai, Songkhla Thailand; 4 Division of Developmental and Behavioral Pediatrics, Department of Pediatrics Faculty of Medicine Prince of Songkla University Hat Yai, Songkhla Thailand; 5 Department of Family and Preventive Medicine Faculty of Medicine Prince of Songkla University Hat Yai, Songkhla Thailand

**Keywords:** generations, logging frequency, mobile health app, weight status, weight reduction

## Abstract

**Background:**

Being overweight or obese presents a major risk factor for noncommunicable diseases (NCDs) such as cardiovascular disease, diabetes, and musculoskeletal disorders. These problems are preventable and solvable via weight reduction and increased physical activity and exercise. The number of adults who are overweight or affected by obesity has tripled in the last 4 decades. Using mobile health (mHealth) apps can help users with health issues, including reducing their weight by restricting their daily calorie intake, which can be recorded along with other parameters, such as physical activity and exercise. These features could further enhance health and prevent NCDs. ThaiSook, a Thai mHealth app developed by the National Science and Technology Development Agency, aims to promote healthy lifestyles and reduce the risk behaviors of NCDs.

**Objective:**

This study aimed to determine whether ThaiSook users were successful in 1-month weight reduction and identify which demographic factors or logging functions were associated with significant weight reduction.

**Methods:**

A secondary data analysis was performed using data collected from the “MED PSU×ThaiSook Healthier Challenge,” a month-long challenge to encourage a healthy lifestyle. We enrolled 376 participants to evaluate the study outcomes. The variables, comprising demographic characteristics (ie, sex, generation, group size, and BMI), were classified into 4 groups: normal (18.5-22.9 kg/m^2^), overweight (23-24.9 kg/m^2^), obese I (25-29.9 kg/m^2^), and obese II (BMI ≥30 kg/m^2^). Logging functions (ie, water, fruit and vegetables, sleep, workout, step, and run) were classified into 2 groups: consistent (≥80%) and inconsistent (<80%) users. Weight reduction was categorized into 3 groups: no weight reduction, slight weight reduction (0%-3%), and significant weight reduction (>3%).

**Results:**

Of 376 participants, most were female (n=346, 92%), had normal BMI (n=178, 47.3%), belonged to Generation Y (n=147, 46.7%), and had a medium group size (6-10 members; n=250, 66.5%). The results showed that 56 (14.9%) participants had 1-month significant weight loss, and the median weight reduction of the group was −3.85% (IQR −3.40% to −4.50%). Most participants (264/376, 70.2%) experienced weight loss, with an overall median weight loss of −1.08% (IQR −2.40% to 0.00%). The factors associated with significant weight reduction were consistently logging workouts (adjusted odds ratio [AOR] 1.69, 95% CI 1.07-2.68), being Generation Z (AOR 3.06, 95% CI 1.01-9.33), and being overweight or being obese compared to those with normal BMI (AOR 2.66, 95% CI 1.41-5.07; AOR 1.76, 95% CI 1.08-2.87, respectively).

**Conclusions:**

More than half of the “MED PSU×ThaiSook Healthier Challenge” participants achieved a slight weight reduction, and 14.9% (56/376) of users lost significant weight. Factors including workout logging, being Generation Z, being overweight, and being obese were associated with significant weight reduction.

## Introduction

Being overweight and being obese are both recognized as major risk factors for noncommunicable diseases (NCDs) such as cardiovascular disease—a leading cause of death—diabetes, and musculoskeletal disorders [1]. These problems are most often preventable and solvable via weight reduction and improvements in physical fitness with increased physical activity and exercise. In 2016, the prevalence of adults who are overweight and affected by obesity globally was 40% and 15%, respectively, a nearly 3-fold increase over the preceding 4 decades [2,3]. Several studies have demonstrated that combining a diet and exercise intervention can help people lose weight more effectively [3,4].

The use of technology to enhance one’s health is no longer a far-fetched concept. It is already used in various contexts, including mobile health (mHealth) apps and wearable technologies [5]. mHealth apps are effective and low-cost tools used to promote health among patients with certain conditions and chronic diseases, including hypertension, obesity, diabetes, and dyslipidemia [2,6-9]. mHealth technology enables changes in health risk behavior, improved patient compliance, and increased self-efficacy [10]. mHealth apps can help users to reduce their weight by restricting their daily calorie intake, which can be recorded along with other parameters, including physical activity and exercise, which could further enhance health and prevent NCDs [11]. A previous study in Korea involving participants who were overweight or affected by obesity indicated that 22.7% of all app users had lost more than 10% of their starting body weight [12]. Another Korean study reported that 55.8% of individuals using the “Noom” app logged their food intake 3 times a day for 16 weeks and lost more weight than those who did not [13]. However, other studies have reported that the use of mHealth apps for weight loss is characterized by a high failure rate, as participants discontinue or limit their usage of the app [14].

The ThaiSook app, a mHealth app developed by the National Science and Technology Development Agency (NSTDA), aims to promote a healthy lifestyle and reduce the health risk behaviors associated with NCDs. The app was used by participants to improve their positive health behaviors through a digital competition model, with expert coaching, team building, and competition between groups to win rewards [15]. According to Life’s Simple 7, based on a publication by Folsom, predictors highly associated with premature death (weight, fruit and vegetable consumption, exercise, blood pressure, blood lipid levels, blood sugar levels, and smoking) [16-18] were recorded in the app. The ThaiSook app is a self-reported program (ie, water consumption, fruit and vegetable consumption, workout, step, run, and sleep logging). ThaiSook can also connect with wearable devices and programs (eg, Xiaomi Scale 2, Apple Health, and Google Fit) and automatically retrieve data (eg, run or step logging). It is widely used by the personnel of 63 Thai hospitals and several health care centers [15].

Despite the current evidence, there are limited studies on the effects of mHealth apps on measurable clinical outcomes, especially weight reduction, in Asia [19]. The results of mHealth interventions on weight reduction were unclear [20]. This study, therefore, aimed to determine whether the participants of the MED PSU×ThaiSook Healthier Challenge could reduce their weight and identify which demographic factors and logging activities were associated with significant weight reduction over 28 days.

## Methods

### Study Design and Setting

An analysis was conducted using secondary data from the ThaiSook database on a prospective cohort that belonged to the NSTDA. The ThaiSook app is easily understood and accessible for Thai individuals of any body weight. The MED PSU×ThaiSook Healthier Challenge, a collaboration between the NSTDA and the Faculty of Medicine, Prince of Songkla University (PSU), encouraged employees to use the app for 28 days. This study was conducted at the Faculty of Medicine, PSU. The “MED PSU×ThaiSook Healthier Challenge” was publicized to the personnel via public posters and social media platforms (ie, Facebook and Line app). Of the 6112 faculty personnel, 827 ThaiSook app users attended and were allowed to assemble teams independently, without exception regarding the number of group members, including one-person teams.

**Figure 1:**
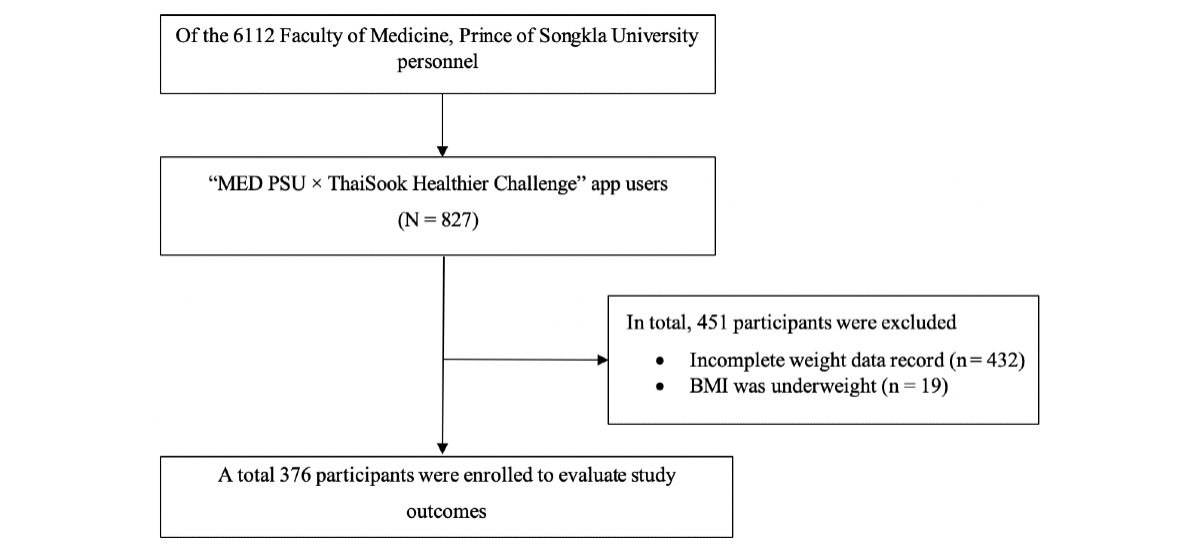
Study flowchart. PSU: Prince of Songkla University.

To meet the inclusion criteria, participants had to be personnel of the Faculty of Medicine, PSU, who engaged in the ThaiSook app between July 11, 2022, and August 7, 2022. The exclusion criteria were as follows: participants who had not completed recording weight data in the ThaiSook app and whose BMI was underweight (n=376); a flowchart of the study is shown in Figure 1.

### Data Collection

#### Overview

**Figure 2:**
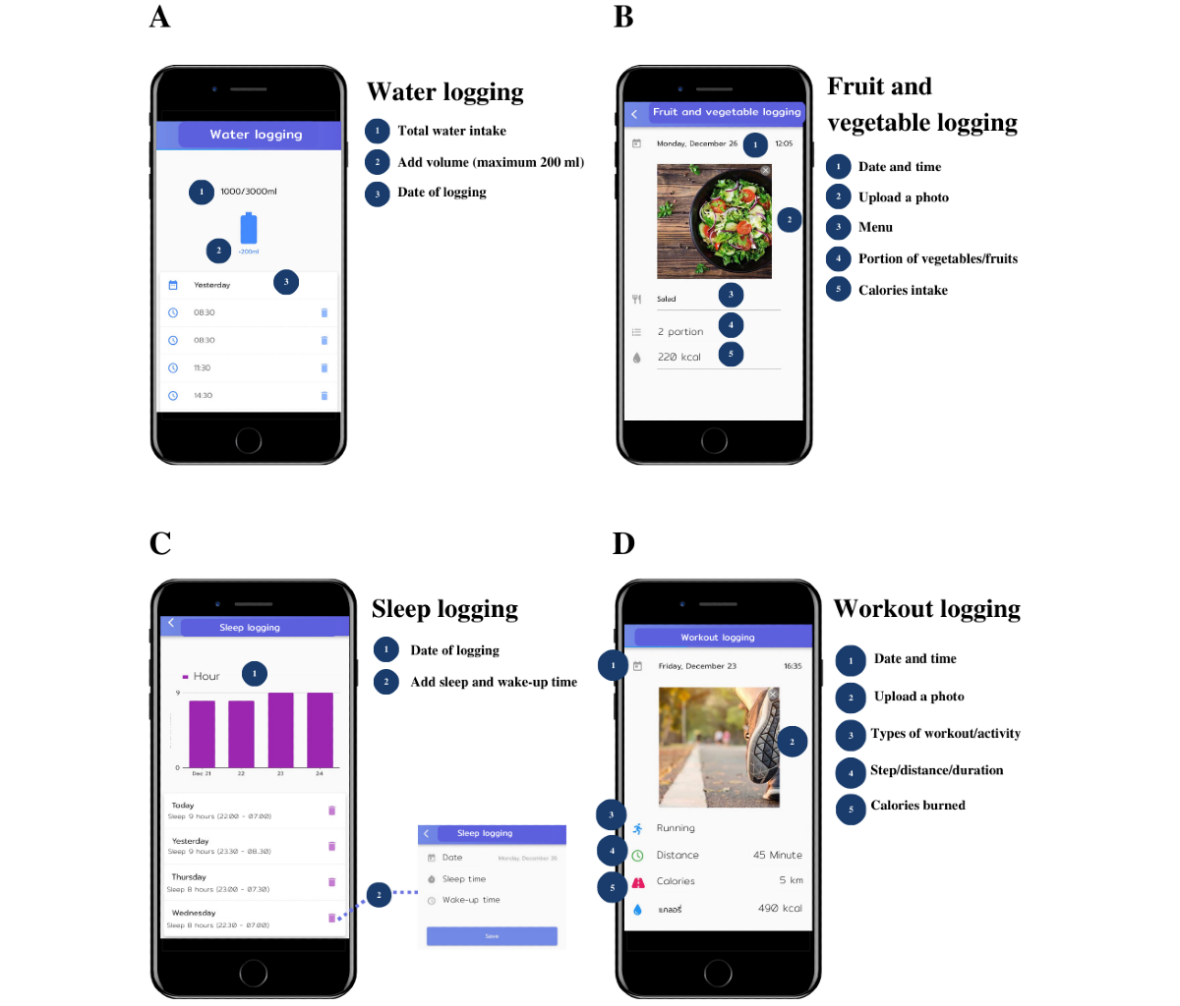
An example of logging functions. (A) water logging, (B) fruit and vegetable logging, (C) sleep logging, and (D) workout logging in the ThaiSook app.

Data were anonymized and extracted using the ThaiSook app developer and sent anonymously with password protection of the data. Data collected from the ThaiSook app included demographic characteristics (ie, age, sex, initial weight, final weight, height, and group size) and the use of the ThaiSook app (logging functions, Table 1 and Figure 2).

**Table 1:** Details of all logging functions used in the ThaiSook app.

Logging functions			Details of each function
Water logging			Record date and drinking time in integer, with a volume limit of 200 mL for each log. For example, drinking water of 400 mL would be 2 logs of 200 mL, counted as 2 records. The highest daily volume reported was 3000 mL.
Fruit and vegetable logging			Upload a photo of food with the menu and log servings of fruit and vegetable with date and time, as well as calories consumed (optional). This function may also be used to log meals without fruits and vegetables.
Sleep logging			Record waking and sleeping times. The app will then determine total sleep time. Users can record more than 1 sleep period per day.
**Workout logging**			Upload a photo and choose the type of activity (ie, stepping, running, walking, cycling, meditation, or others) and note the intensity of each activity type (ie, step, duration, and distance), calculated as calories burned (optional). There is different recording information for each activity type.
	Step logging	Upload a photo and log the number of steps in a day (only the last recorded steps will be logged that day), distance (optional), and calories burned (optional).	
	Run logging	Upload a photo and log running time (minutes), distance (optional), and calories burned (optional).	
App use			Use logging features to keep track of daily activities (ie, exercise, food and water consumption, sleep hours, weight, blood pressure, and blood test results). The app will then show a summary of usage and overall health behaviors. As part of the study, there was a virtual competition in which people could compete in teams.

#### Independent Variables—Demographic Characteristics and Functions Use

The demographic data comprised information on sex, generation, BMI, and group size. Generations (age) were classified into 4 groups: Baby Boomers (born from 1946 to 1964; current age 58-76 years), Generation X (born 1965-1980; current age 42-57 years), Generation Y (born 1981-1996; current age 26-41 years), and Generation Z (born 1997-2012; current age 10-25 years) [21]. BMI was classified according to the World Health Organization (WHO) classification of BMI for Asian adults into normal (18.5-22.9 kg/m^2^), overweight (23-24.9 kg/m^2^), obese I (25-29.9 kg/m^2^), and obese II (BMI ≥30 kg/m^2^) [22]. There were 4 different group sizes: individual (1 person per group), small group (2-5 people per group), medium group (6-10 people per group), and large group (>10 people per group).

The MED PSU×ThaiSook Challenge was conducted for 28 days. The number of days the app was used serves as a measure of user participation. We categorized ThaiSook app use into two groups according to the frequency of app logging.

1. Consistent users: participants who logged at least 1 function in the app ≥80% of the time (≥23 days)
2. Inconsistent users: participants who logged at least one function in the app <80% of the time (<23 days).

#### Dependent Variables—Weight Reduction Percentage

Participants’ body weights (kg), which comprised self-reported initial body weight on the first day and final body weight on day 28 of the challenge, were logged on the ThaiSook app. Body weight changes after the 28-day challenge were categorized into three groups.

1. No weight reduction: Participants whose body weight at the end of the 28-day challenge was equal to or greater than their initial body weight
2. Slight weight reduction: participants whose body weight at the end of the 28-day challenge decreased by less than 3% of their initial body weight
3. Significant weight reduction: participants whose body weight at the end of the 28-day challenge decreased by 3% or more of their initial body weight

According to a Japanese study by Muramoto et al [23], weight reduction above 3% is the minimum required for the improvement of all parameters for obesity-related diseases (ie, plasma triglycerides, low-density lipoprotein cholesterol, hemoglobin A_1c_, aspartate aminotransferase, alanine aminotransferase, γ-glutamyl transpeptidase, high-density lipoprotein cholesterol, systolic blood pressure, diastolic blood pressure, fasting plasma glucose, and uric acid, a total of 11 parameters) in the Asian population. Therefore, our study used weight reduction of at least 3% as the cutoff for determining considerable health outcome improvement after the 28-day challenge.

### Power Analysis

A post hoc power analysis was performed, and a sample size of 376 was used for statistical power analysis. The alpha level used in the analysis was *P*<.05. The post hoc analysis revealed that the power for combining each BMI value exceeded 0.97, and the same was observed for workout logging function use and generation.

### Statistical Analysis

Statistical analysis was performed using *R* software (version 4.2.1; R Foundation for Statistical Computing) with data.table, rio, epicalc, table1, and readxl packages (Multimedia Appendix 1). Descriptive statistics were presented as medians with IQR and percentages and were used for demographic characteristics and app logging. The characteristics of the weight reduction group were compared using a Mann-Whitney *U* test, a Kruskal-Wallis test for continuous variables, and a Pearson chi-square test for categorical variables. A multiple ordinal logistic regression was performed to identify the variables associated with weight reduction outcomes (ie, no weight reduction as a reference vs slight weight reduction vs significant weight reduction). Various variables (*P*<.20) were obtained in the univariate analysis, and potential independent variables included sex, generation (age), BMI, and workout logging. A manual backward stepwise refinement was performed for the final model. Adjusted odds ratios (AORs) and 95% CIs were also calculated. Furthermore, a *P*<.05 was considered statistically significant.

### Ethics Approval

The clinical trial was registered according to the WHO International Clinical Trials Registry Platform at the Thai Clinical Trials Registry with registry ID TCTR20220611001. This study was approved by the Human Research Ethics Committee of the Faculty of Medicine, PSU (REC.65-395-9-2). Informed consent was obtained for the primary data collection. The Human Research Ethics Committee allows additional informed consent to be waived from the secondary analysis. All information collected from the participants was kept confidential, anonymous, and accessible only to the researchers through the ThaiSook database. No compensation was provided.

## Results

### Baseline Demographic Characteristics of Study Participants

The 827 ThaiSook app users who participated in the “MED PSU×ThaiSook Healthier Challenge” and who fulfilled the inclusion criteria were reviewed. We excluded 451 participants based on the exclusion criteria (432 participants with incomplete body weight data in the ThaiSook app and 19 participants who were underweight). Finally, 376 participants were included in the analysis to evaluate the study outcomes.

The baseline characteristics of the 376 participants are shown in Table 2. Most participants were female (346/376, 92%), had a normal BMI (178/376, 47.3%), and belonged to Generation Y (147/376, 46.7%). Most participants were also in the medium group size (6-10 people; n=250, 66.5%).

**Table 2:** Demographic characteristics by weight reduction after the 28-day challenge (n=376).

Characteristics			Participants, n (%)	Values, median (IQR)	No weight reduction (weight gained-0%), n (%)	Slight weight reduction (0%-3%), n (%)	Significant weight reduction (≥3%), n (%)
Total			376 (100)	—^a^	112 (29.8)	208 (55.3)	56 (14.9)
Total, median (IQR)		—		−1.08 (−2.40 to 0.00)	0.70 (1.40 to 0.15)	−1.49 (−0.87 to −2.18)	−3.85 (−3.40 to −4.50)
**Sex**							
	Male		30 (8)	−2.00 (−3.68 to −0.53)	6 (20)	13 (43)	11 (37)
	Female		346 (92)	−1.03 (−2.33 to 0.00)	106 (30.6)	195 (56.4)	45 (13)
	*P* value		—	.73^b^	—	—	.002^c,d^
**Group size**							
	1		3 (0.8)	–0.19 (−0.19 to −0.09)	1 (33)	2 (67)	0 (0)
	2-5		97 (25.8)	−1.28 (−2.67 to 0.00)	25 (26)	54 (56)	18 (18)
	6-10		250 (66.5)	−1.07 (−2.38 to 0.00)	75 (30)	142 (56.8)	33 (13)
	≥11		26 (6.9)	−0.79 (−2.02 to 0.74)	11 (42)	10 (38)	5 (19)
	*P* value		—	.49^b^	—	—	.42^e^
**Generation**							
	Baby boomers		19 (6)	−0.84 (−1.28 to 0.55)	8 (42)	9 (47)	2 (10)
	Generation X		135 (42.9)	−1.01 (−2.21 to 0.00)	43 (32)	78 (58)	14 (10)
	Generation Y		147 (46.7)	−1.01 (−2.57 to 0.00)	41 (28)	81 (55)	25 (17)
	Generation Z		14 (4.4)	−2.55 (−3.04 to −1.11)	2 (14)	8 (57)	4 (29)
	*P* value		—	.45^b^	—	—	.26^e^
**Initial BMI**							
	Normal		178 (47.3)	−0.67 (−2.04 to 0.34)	69 (39)	87 (49)	22 (12)
	Overweight		64 (17)	−1.61 (−2.81 to −0.91)	11 (17)	40 (62)	13 (20)
	Obese I		115 (30.6)	−1.47 (−2.44 to −0.14)	28 (24)	67 (58)	20 (17)
	Obese II		19 (5)	−1.40 (−2.68 to 0.46)	4 (21)	14 (74)	1 (5)
	*P* value		—	.46^c^	—	—	.01^c,d^

^a^Not applicable.

^b^Mann-Whitney *U* test.

^c^Kruskal-Wallis test.

^d^*P* value <.05.

^e^Pearson chi-square test.

### Impact of Demographic Characteristics on Weight Reduction

As shown in Table 2, 70.2% (264/376) of participants were able to reduce weight, and 14.9% (56/376) reported a significant weight reduction (more than 3%). The overall median weight loss was −1.08% (IQR−2.40 to 0.00), while for the significant weight reduction group, it was −3.85% (IQR −3.40 to −4.50). After 28 days, 37% (11/30) of the male participants showed significant weight reduction, while only 13% (45/346) of the female participants showed significant weight loss. Notably, 29% (4/14) of Generation Z showed significant weight reduction, followed by 17% (25/147) of Generation Y. Moreover, 20% (13/64) of the individuals whose BMI fell into the overweight category showed significant weight reduction, followed by the individuals whose BMI fell into the obese I (17.4%) category, while only 5.3% of those whose BMI fell into the obese II category showed significant weight loss. The univariate analysis showed that sex and initial BMI were significantly associated with weight loss when comparing the 3 weight reduction groups (*P*<.05). Demographic characteristics (ie, sex, initial BMI, generation, and group size) were not found to be significantly associated with weight reduction when compared with the median weight loss.

### Logging Functions of the ThaiSook App and Its Influence on Weight Reduction

**Figure 3:**
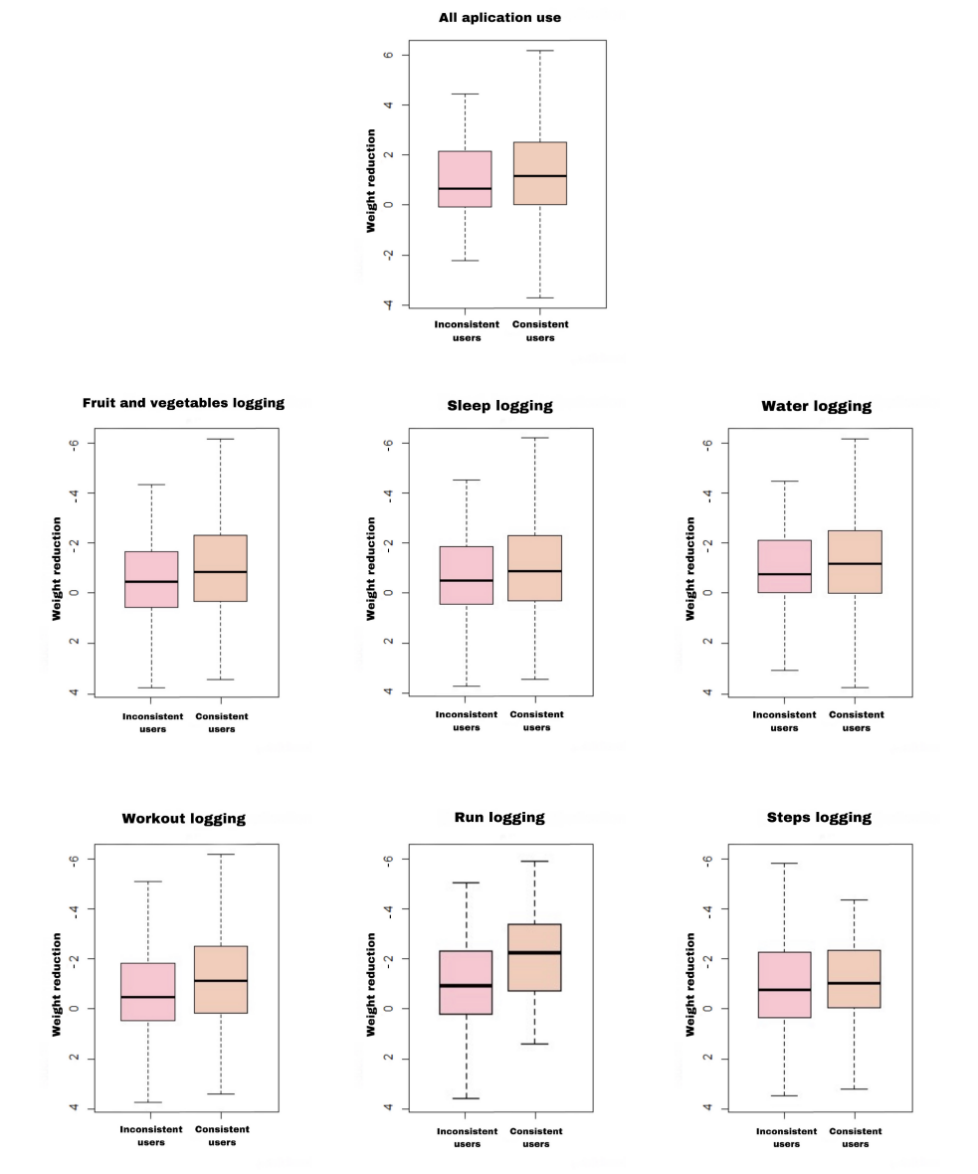
Distribution median of weight reduction by logging functions of ThaiSook app in 28 days.

According to Table 3, consistent users (≥80%) of the ThaiSook app reduced their weight by 73.3% (211/288), while inconsistent users (<80%) had weight reductions of 60.2% (53/88). However, the univariate analysis indicated no significant association between each logged function of the app (ie, app use, water, fruit and vegetable, sleep, workout, step, and run logging) and the 3 weight reduction groups. When comparing the median weight reduction, the consistent users had significantly greater scores than inconsistent users in terms of app use, fruit and vegetable, sleep, water, and workout logging (*P*<.05), as illustrated in Figure 3.

**Table 3:** Distribution of weight reduction by logging functions of ThaiSook app use in 28 days.

Function of app			Participants, n (%)	Value, median (IQR)	No weight reduction (weight gained-0%), n (%)	Slight weight reduction (0%-3%), n (%)	Significant weight reduction (≥3%), n (%)
**App use**							
	Inconsistent user		88 (23)	−0.49 (−2.14 to 0.00)	35 (40)	38 (43)	15 (17)
	Consistent user		288 (76.6)	−1.12 (−2.51 to 0.00)	77 (27)	170 (59)	41 (14)
	Total		376 (100)	−1.08 (−2.40 to 0.00)	112 (29.8)	208 (55.3)	56 (15)
	*P* value		—^a^	<.001^b,c^	—	—	.26^d^
**Fruit and vegetable logging**							
	Inconsistent user		148 (39.7)	−0.89 (−2.06 to −0.14)	52 (35)	76 (51)	20 (13)
	Consistent user		255 (68.3)	−1.24 (−2.60 to 0.00)	58 (26)	131 (58.2)	36 (16)
	Total		373 (100)	−1.09 (−2.39 to 0.00)	110 (29.4)	207 (55.5)	56 (15)
	*P* value		—	<.001^b,c^	—	—	.15^d^
**Sleep logging**							
	Inconsistent user		162 (44.9)	−0.90 (−2.24 to 0.00)	57 (35)	81 (50)	24 (15)
	Consistent user		199 (55.1)	−1.24 (−2.57 to 0.00)	51 (26)	119 (60)	29 (14.6)
	Total		361 (100)	−1.07 (−2.40 to 0.00)	108 (29.9)	200 (55.4)	53 (15)
	*P* value		—	<.001^b,c^	—	—	.12^d^
**Water logging**							
	Inconsistent user		108 (28.7)	−0.82 (−2.17 to 0.00)	39 (36)	51 (47)	18 (17)
	Consistent user		268 (71.3)	−1.17 (−2.53 to 0.00)	73 (27)	157 (58.6)	38 (14)
	Total		376 (100)	−1.08 (−2.40 to 0.00)	112 (29.8)	208 (55.3)	56 (15)
	*P* value		—	<.001^b,c^	—	—	.13^d^
**Workout logging**							
	Inconsistent user		208 (59.4)	−0.89 (−2.17 to 0.00)	67 (32)	116 (55.8)	25 (12)
	Consistent user		142 (40.6)	−1.49 (−2.78 to −0.23)	33 (23)	83 (58)	26 (18)
	Total		350 (100)	−1.09 (−2.48 to 0.00)	100 (28.6)	199 (56.9)	51 (14)
	*P* value		—	.02^b,c^	—	—	.09^d^
	**Step logging**						
		Inconsistent user	215 (86.3)	−1.05 (−2.54 to 0.20)	66 (31)	114 (53)	35 (16)
		Consistent user	34 (14)	−1.39 (−2.58 to −0.39)	7 (21)	23 (68)	4 (12)
		Total	249 (100)	−1.09 (2.58 to 0.00)	73 (29)	137 (55)	39 (16)
		*P* value	—	.99^b^	—	—	.28^d^
	**Run logging**						
		Inconsistent user	250 (87.7)	−1.03 (−2.37 to 0.00)	72 (29)	144 (57.6)	34 (14)
		Consistent user	35 (12)	−1.92 (−2.94 to 0.55)	8 (23)	19 (54)	8 (23)
		Total	285 (100)	−1.09 (−2.51 to 0.00)	80 (28)	163 (57)	42 (15)
		*P* value	—	.74^b^	—	—	.33^d^

^a^Not applicable.

^b^*P* value <.05.

^c^Mann-Whitney U test.

^d^Pearson chi-square test.

### Factors Associated With a More Than 3% Weight Reduction

Tables 2 and 3 show that the univariate model revealed factors such as sex (*P*=.002), BMI (*P*=.01), and app use (*P*=.03) to be associated with the three weight reduction groups. After adjusting for potential confounders (ie, group size, fruit and vegetable logging, sleep logging, and water logging), an ordinal logistic regression was used to identify factors associated with significant weight reduction. Generation Z had 3.06 times higher significant weight reduction than Generation Y (AOR 3.06, 95% CI 1.01-9.33). Moreover, being overweight and being obese were more strongly associated with weight reduction than normal BMI (AOR 2.66, 95% CI 1.41-5.07; AOR 1.76, 95% CI 1.08-2.87). The consistent users (≥80%) of workout logging reported significant weight reduction (1.69 times) when compared to inconsistent users (<80%) (AOR 1.69, 95% CI 1.07-2.68), as presented in Table 4.

**Table 4:** Ordinal logistic regression of significant weight reduction with demographic characteristics and app logging functions.

Characteristics		Adjusted OR^a^ (95% CI)	*P* value
**Sex (Ref^b^=Female)**			
	Male	2.31 (1.00-5.36)	.05
**Generation (Ref=Generation Y)**			
	Baby boomers	0.46 (0.17-1.19)	.12
	Generation X	0.68 (0.42-1.08)	.11
	Generation Z	3.06 (1.01-9.33)	.048^c^
**Initial BMI (Ref=Normal)**			
	Overweight	2.66 (1.41-5.07)	.02^c^
	Obesity	1.76 (1.08-2.87)	.003^c^
**Workout logging (Ref≤80%)**			
	≥80%	1.69 (1.07-2.68)	.03^c^

^a^OR: odds ratio.

^b^Ref: reference.

^c^*P* value <.05.

## Discussion

### Principal Findings and Previous Studies

The study revealed that, throughout the 28-day challenge, 56 (15%) of the 376 ThaiSook app users had significant weight reduction (more than 3%), and 264 (70.2%) achieved weight reduction. This finding may be explained by the fact that ThaiSook users could reduce their weight by using any logging function on the app to help them limit their daily diet and keep track of several parameters, including their physical activity and food consumption. However, due to the competition effect, the proportion of participants who experienced weight reduction in this study may have been overestimated compared to what it would have been in a normal situation [24]. This result might be explained by the environment created by the “MED PSU×ThaiSook Healthier Challenge” to maintain a competitive system. The various rewards for successful weight loss are also viewed as major motivators that encouraged app usage. Our results are consistent with those of a Korean study on the Noom Coach Health App, in which 77.9% of users reported that their body weight decreased while using the program. However, 22.7% of Noom app users reported losing more than 10% of their initial weight, which is more than what was lost in our study [13]. This may be because, in that study, the Noom app was used for a longer period (6 months), while in our study, the app was used only for a short period (28-day challenge) [13]. Our study used ≥3% weight loss as a cutoff for the main study outcome; thus, the cut-off in our study is appropriate. Regarding ThaiSook app usage, our study found that 288 (76.6%) of participants consistently (≥80%) accessed the app. The support provided by group members and competitive rewards for successful weight loss are considered significant motivators that encourage ThaiSook app usage.

### Predicting Factors of Significant Weight Reduction After the 1-Month Challenge

The ordinal logistic regression identified 4 elements significantly associated with substantial weight reduction: belonging to Generation Z, being overweight, being obese, and consistently using a workout log.

Our results revealed that over the 28 days, being from Generation Z was related to significant weight reduction compared with Generation Y. This difference could result from the nature and social conditions of the generations, as well as the relationship between generational concepts and health apps. According to previous studies, each generation has a different preference for mHealth apps and wearable devices. For example, Fox and Connolly [25] reported that older generations tend to display modest health app use; they are reluctant to use them due to mistrust, a high perception of risk, and a strong desire for privacy, considering the protection motivation theory and social cognitive theory. Conversely, younger generations are highly dependent on mobile devices and have a greater potential to use mHealth apps [26]. Previous research supports this theory by suggesting that younger generations, Generations Y and Z, can use and adapt to technology better than older generations [27]. Although most members of Generation Z reported using mHealth apps at least once in their lifetime, only a minority have used the app in this study [28,29]. However, Generation Y uses significantly more wearable health devices than the other generations [30]. This tendency suggests that different generations have different concerns regarding the use of mHealth technology in improving health.

Although there is evidence that an individual’s generation plays a role in their use of mHealth apps and wearable devices, there is a lack of evidence on the outcome of mHealth app use, such as weight reduction, between generations. Furthermore, the concept of body image may help explain the relationship between weight reduction and generation. Younger generations are more self-conscious of their bodies than the previous generations. A previous study examined body image across 3 generations and found that younger people—aged 17-21 years—were more dissatisfied with their bodies than older generations [31]. Therefore, there might be a relationship between younger generations, Generation Z, and weight reduction, as the perception of body image differs across generations. Moreover, among Generation Z individuals (current age 10-25 years), almost all participants were adolescents and early adults; this enables them to lose significantly more weight in 1 month than older age groups, as age and metabolic rate are connected. Increased age leads to a nearly linear decline in basal metabolic rate [32,33]. Furthermore, Generation Z has been shown to be more eager to implement positive changes in their lives and to cherish their health [34].

According to our results, participants who were overweight or being obese proportionally lost more body weight than those with normal BMI. This association might be explained by the body’s metabolic rate concept: the body’s metabolic rate increases along with an increase in body weight [32,35]. Therefore, individuals who are overweight or being obese could quickly lose more weight than those with normal or low BMI while consuming the same amount of energy and exercising equally. However, the groups of individuals who were overweight or obese also differed, and the proportion of weight reduction in the group of individuals who were overweight was greater than in the group of individuals who were obese (2.66 vs 1.76) compared with the normal BMI group. This observation is likely related to the stage of change theory and the obesity “point of no return,” according to which individuals who are obese and have a BMI of 30 kg/m^2^ or greater found that maintaining weight loss was rare and the probability of achieving normal weight extremely low, making weight loss difficult [36]. Additionally, we included participants with normal BMI in the “MED PSU×ThaiSook Healthier Challenge” to determine the association between using mHealth apps and significant weight reduction. Previous studies indicated that weight loss was advantageous for those with normal BMI, particularly in terms of reducing nonalcoholic fatty liver disease [37], blood pressure [38], and risk of stroke [39].

Considering the workout logs, the participants who consistently logged their workouts showed significant weight loss compared with those who logged inconsistently. This may explain why too much of the public’s understanding, exercise, and physical activity are directly related to weight loss. A previous study revealed that individuals in the high-exercise group lost more weight than the low-exercise group [40]. Therefore, participants who were high-exercise or had high physical activity could record run logging and step logging in the ThaiSook app. Moreover, the workout logging function is also easier to use compared to other logging functions as it can be connected with existing wearable devices or other health apps, and users can transfer exercise or physical activity data (ie, step and running) from other devices to the ThaiSook app. In addition, workout logging was an amalgamation of 2 subfunctions, step logging and run logging, which consisted of 2 consistent user groups that tracked their workouts—individuals who ran for exercise and those who walked. This may be why weight loss participants often used this logging function.

### Strengths and Limitations

This study is one of the few app-based studies that collected real-world data on weight loss and health effects with mHealth app usage conducted in upper-middle income countries [41]. This study aimed to determine the association between measurable clinical outcomes, such as significant weight reduction, with each logging function of the app and demographic variables. Our study investigated ThaiSook app usage directly from behavior (logging) and did not collect data from questionnaires, which might introduce information or recall bias.

Our most significant limitation was data collection, as we used convenience sampling to distribute the “MED PSU×ThaiSook Healthier Challenge.” Most of the participants were female and aged between 26 and 57 years. However, the proportion of sex and age groups of participants corresponded to the proportion of faculty personnel. Therefore, our study might have limited external validity, and the findings cannot easily be generalized to general populations or organizations with characteristics that differ from those of these participants. Additionally, most of our study participants who used the ThaiSook app were employed in health care organizations. Hence, their health knowledge and literacy may be superior to those of the general population. However, this outcome could be a result of the target demographic being concentrated in a health care workplace where women outnumber men, such as a hospital.

Second, our study was conducted over a short period—1 month. This contrasts with previous weight loss app studies in Korea; these studies considered participants who were overweight or being obese and indicated that the median time spent using an app was 267 days [11]. This study found that they could lose significant weight more quickly compared to others. Recognizing this could encourage patients to maintain weight loss for extended periods. Therefore, further studies should extend the study period to 3 or 6 months to more effectively determine the effectiveness of health apps and measures for significant weight reduction and maintaining body weight.

Third, users provided inconsistent weight tracking, particularly their final weight, and the extent of this missing data may be significant. Most app users who took part were excluded from the analysis due to incomplete records of final weight at the end of the 28 days of using ThaiSook app. Furthermore, the users’ desired use period was unrelated to the 28-day research period. Consequently, their final weight was not documented 28 days after the start of ThaiSook.

Finally, the storage process is inadequate. Developers should establish clear and simple filling forms for ThaiSook users to avoid confusion and errors when entering information. The accuracy of the measuring instruments used by ThaiSook users to weigh themselves and determine their weight and height is disputed. Due to nonstandard results, they cannot be compared between groups or individuals. In most cases, reports of a person’s weight and height are biased [42]. They also typically reported underweight outcomes and a greater height. Additionally, some logging features, especially sleep logging, were lacking. Sleep logging only recorded the user's bedtime and wake-up time, not how long they took to fall asleep once in bed, which could potentially introduce information bias.

### Implication and Further Studies

This study highlights the potential of mobile health apps, specifically the ThaiSook app, in promoting healthy lifestyles and reducing the risk of NCDs. The results suggest that consistent workout logging, being part of the Generation Z age group, and being overweight or being obese are associated with significant weight reduction among app users. These findings underscore the importance of tracking physical activity and targeting specific demographic groups when promoting weight loss through mHealth interventions.

To improve the findings of this study, the target population should be more diverse, and data collection should be conducted using standardized methods and measures. For example, measurable health data should be collected from wearable devices, such as smartwatches, rather than self-reported data. In addition, extending the period to 3 or 6 months—rather than only 28 days—could yield more significant results on sustained weight loss and provide more perspectives.

### Conclusions

The results of this study revealed that not all users of the app could reduce their weight. However, we do provide some remarkable results, namely that about 14.9% of ThaiSook app users were able to achieve significant weight reduction, while 55.3% of users achieved a slight weight reduction. This study also discovered that certain characteristics, such as logging workouts, belonging to Generation Z, and being overweight or being obese, are associated with considerable weight reduction.

## Appendix

Multimedia Appendix 1 R software packages and R codes.

## Abbreviations

AOR: adjusted odds ratio
mHealth: mobile health
NCDs: noncommunicable diseases
NSTDA: National Science and Technology Development Agency
PSU: Prince of Songkla University
Ref: reference
WHO: World Health Organization

## Data Availability

The data sets used and analyzed during this study are available from the corresponding author upon reasonable request.
