# Supplementary material for: Workout Logging Through an mHealth App for Weight Reduction Among Different Generations: Secondary Analysis of the MED PSU×ThaiSook Healthier Challenge
Source: JMIR Form Res. 2023 Jun 30;7:e45298. doi: 10.2196/45298 (PMC10365584; doi:10.2196/45298)
Supplement: Multimedia Appendix 1 [file formative_v7i1e45298_app1.pdf]

Supplement 1

Code

```
setwd("D:/Thaisook")

lumpuk4<-setDT(import("lumpuk4.csv"))

names(lumpuk4)

lumpuk4[,.N,category]

lumpuk4[,.N,menu]

lumpuk4[,.N,.(endWeightVerify,uid,home)]

mm<-lumpuk4[,.N,.(endWeightVerify,uid,home)]

mm[,.N,endWeightVerify]


lumpuk4[,.N,.(endWeightVerify,uid,home,startWeight,endWeight,category,menu,)]

lumpuk4[,weightdiff:=startWeight-endWeight]

lumpuk4[,.N,.(uid,home,weightdiff)]

lumpuk4[,.N,weightdiff][order(weightdiff)]

use(lumpuk4)

summ(weightdiff)

names(lumpuk4)

lumpuk4<-lumpuk4[endWeightVerify==TRUE]

lumpuk4[,.N,.(uid,home,endWeightVerify)]

summ(weightdiff)

lumpuk4[,perwtreduct:=(weightdiff/startWeight)*100]

lumpuk4[,.N,.(uid,home,weightdiff,perwtreduct)][order(perwtreduct)]

use(lumpuk4)

summ(perwtreduct)

boxplot(perwtreduct)

names(lumpuk4)

lumpuk4[,sucsess:=ifelse(perwtreduct>=3,">=3",

            ifelse(perwtreduct>0,"0-3","<=0"))]

lumpuk4[,.N,.(uid,home,perwtreduct,sucsess)][order(perwtreduct)]

lumpuk4[,.N,sucsess]
```

```
lumpuk5<-setDT(import("lumpuk5.csv"))

lumpuk5->lumpuk4

lumpuk4[,.N,.(uid,home)]

lumpuk4[,.N,uid]

##gender-frequency

lumpuk4[,.N,.(uid,home,gender)]

tt<-lumpuk4[,.N,.(uid,home,gender)]

names(tt)[4]<-"Frequency"

tt

use(tt)

summ(Frequency)

tt[,.N,gender]

tt[,uol:=ifelse(Frequency>=255,"maak","noi")]

tt

tt[,.N,.(gender,uol)]

use(tt)

tabpct(gender,uol)

tableStack(c(gender),by=uol,total.column = TRUE,percent = "row")

anda<-tableStack(c(gender),by=uol,total.column = TRUE,percent = "row")

##write.csv(anda,"anda.csv")


##Gender-Duration

#Duration generation

rm(list = ls())

lumpuk5<-setDT(import("lumpuk5.csv"))

names(lumpuk5)

lumpuk5[,c(1,29,6,3)]

dd<-lumpuk5[,c(1,6,3)]

dd

dd[,.N,.(date,uid,gender)]

ss<-dd[,.N,.(date,uid,gender)]

ss<-ss[,1:3]

ss
```

```
ss<-ss[order(uid)]

ss

ss[,.N,.(uid,gender)]

ss[,.N,.(uid,gender)][order(N)]

xx<-ss[,.N,.(uid,gender)][order(N)]

xx

names(xx)[3]<-"frequency"

xx

summ(xx$frequency)

shapiro.test(xx$frequency)

boxplot(xx$frequency)

xx[,flevel:=ifelse(frequency>=14,"maak","noi")]

use(xx)

tableStack(c(gender),by=flevel,total.column = TRUE,percent = "row")

tab01genderduration<-tableStack(c(gender),by=flevel,total.column = TRUE,percent = "row")


#write.csv(tab01genderduration,"tab01genderduration.csv")


#gender-daily

zz<-xx

zz

zz[,flevel:=ifelse(frequency>=28,"tukwan","maitukwamn")]

zz

use(zz)

tableStack(c(gender),by=flevel,total.column = TRUE,percent = "row")

tab01genderdaily<-tableStack(c(gender),by=flevel,total.column = TRUE,percent = "row")


#write.csv(tab01genderdaily,"tab01genderdaily.csv")
```
